# Population structure and connectivity of tiger sharks (*Galeocerdo cuvier*) across the Indo-Pacific Ocean basin

**DOI:** 10.1098/rsos.170309

**Published:** 2017-07-05

**Authors:** Bonnie J. Holmes, Samuel M. Williams, Nicholas M. Otway, Einar E. Nielsen, Safia L. Maher, Mike B. Bennett, Jennifer R. Ovenden

**Affiliations:** 1 School of Biomedical Sciences, The University of Queensland, St Lucia, Queensland 4072 Australia; 2 Molecular Fisheries Laboratory, The University of Queensland, St Lucia, Queensland 4072 Australia; 3 New South Wales Department of Primary Industries, Port Stephens Fisheries Institute, Taylors Beach, New South Wales 2316, Australia; 4 National Institute of Aquatic Resources, Technical University of Denmark, Vejlsøvej 39, 8600 Silkeborg, Denmark

**Keywords:** tiger shark, *Galeocerdo cuvier*, population structure, microsatellite loci, Indo-Pacific Ocean

## Abstract

Population genetic structure using nine polymorphic nuclear microsatellite loci was assessed for the tiger shark (*Galeocerdo cuvier*) at seven locations across the Indo-Pacific, and one location in the southern Atlantic. Genetic analyses revealed considerable genetic structuring (*F*_ST_ > 0.14, *p* < 0.001) between all Indo-Pacific locations and Brazil. By contrast, no significant genetic differences were observed between locations from within the Pacific or Indian Oceans, identifying an apparent large, single Indo-Pacific population. A lack of differentiation between tiger sharks sampled in Hawaii and other Indo-Pacific locations identified herein is in contrast to an earlier global tiger shark nDNA study. The results of our power analysis provide evidence to suggest that the larger sample sizes used here negated any weak population subdivision observed previously. These results further highlight the need for cross-jurisdictional efforts to manage the sustainable exploitation of large migratory sharks like *G. cuvier*.

## 1. Introduction

Migratory species are described as entire populations (or any geographically separate part of a population), whose members cyclically and predictably cross one or more national jurisdictional boundaries [1]. In the marine environment, defining sharks as migratory species requires considerable knowledge of individual movement patterns, as well as an understanding of contemporary population structure. As with all wild populations, the population structure of sharks is determined in genetic terms by genetic drift, mutation and selection [2,3]. Countering the influence of these microevolutionary forces is gene flow, which mediates genetic isolation through the successful reproduction of immigrants *in situ* [4]. In highly migratory marine species, the expectation is that signatures of genetic differentiation are less pronounced, as their large dispersal capabilities permit ongoing population connectivity, even at a global scale [5]. However this is not always the case, with evidence of genetic structuring for some migratory shark species at regional geographical scales, due to the restriction of gene flow by large oceanic expanses, phylogeographic boundaries, or thermal barriers [3,6–9]. Identifying these subpopulations within the broader distribution of migratory sharks is a critical step in delineating the stocks, which need to be identified for potential conservation and management of the species [3].

The tiger shark (*Galeocerdo cuvier*) (Péron and Lesueur, 1822) is the largest of the Carcharhiniformes, with a circumglobal distribution in tropical and warm temperate coastal and pelagic waters. Across the Indo-Pacific Ocean basin tiger sharks are targeted by a range of commercial, recreational, artisanal and shark control fishing operations [10–14]. Despite these pressures, little is known about the population size throughout this region, with long-term catch rate trends providing the only potential estimate of abundance to date [15]. Satellite tracking studies across the Indo-Pacific have documented round trip migrations of over 5000 km, with individuals tagged off the Australian east coast recording movements across the Coral Sea to New Caledonia [16,17] and Papua New Guinea [18]. Research based in Western Australia has also shown broad-scale movements into the Northern Territory and Indonesian waters [19], and potentially across the broader Indian Ocean [20]. In contrast to these broad movement patterns, tiger sharks tagged off Hawaii appear to exhibit more restricted movements within the Hawaiian island group [21–23]. A feature of all of these studies is the return of tiger sharks to specific areas on a regular basis, which has thus far been attributed to seasonal feeding excursions rather than fidelity to mating or parturition grounds, the latter of which has not yet been identified in this species. Indeed, the ability to identify philopatry and sex-biased dispersal through satellite tracking studies of tiger sharks has been hampered by the need to capture and tag adequate numbers of animals from both sexes across their full size range. Further, as female tiger sharks are likely to have a triennial reproductive cycle in the Pacific [24], identifying a return to natal grounds would require several years of satellite tracking data, which remains problematic due to restrictions on tag battery life, retention and biofouling.

Different management and monitoring regimes of neighbouring jurisdictions, coupled with a lack of fishing regulation in international waters, generate significant threats to migratory shark species [25]. For tiger sharks this is of particular concern, with a recent global study by Bernard *et al*. [9] reporting nDNA genetic differentiation between western Atlantic and Indo-Pacific Ocean basins, indicating wide-ranging populations that inhabit and move through a variety of fishing jurisdictions, Exclusive Economic Zones (EEZ) and international waters. Bernard *et al*. [9] also suggested there may be limitations to the connectivity of tiger sharks across the central Pacific Ocean, with the tiger sharks sampled in the Hawaiian archipelago assigned to a separate population.

Using nine nDNA microsatellite loci previously developed for the species [26], the current study focuses on tiger shark population connectivity and dynamics in the Indo-Pacific region. Specifically, our aims were to: (i) detect population genetic structuring across the eastern Indian and central and western Pacific Oceans using large sample sizes to provide good statistical power, and (ii) test the expectation that tiger sharks from Hawaii assign to a separate population from the broader Indo-Pacific region.

## 2. Methods

### 2.1. Sample collection, extraction methods and genotyping

**Figure 1.**
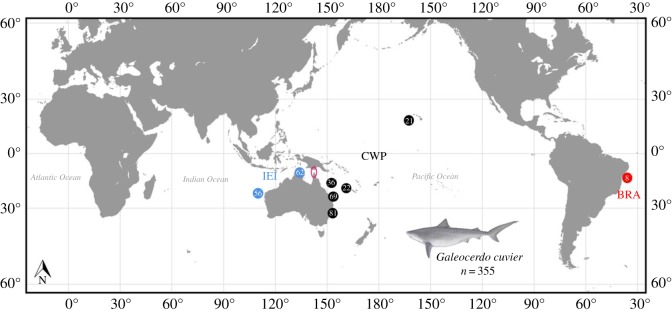
Sampling locations and number of *G. cuvier* tissue samples obtained from the Indo-East Indian (IEI, blue), the Central-West Pacific (CWP, black) and the Atlantic Ocean (BRA, red). Pink open circle denotes the Torres Strait land bridge.

**Figure 2.**
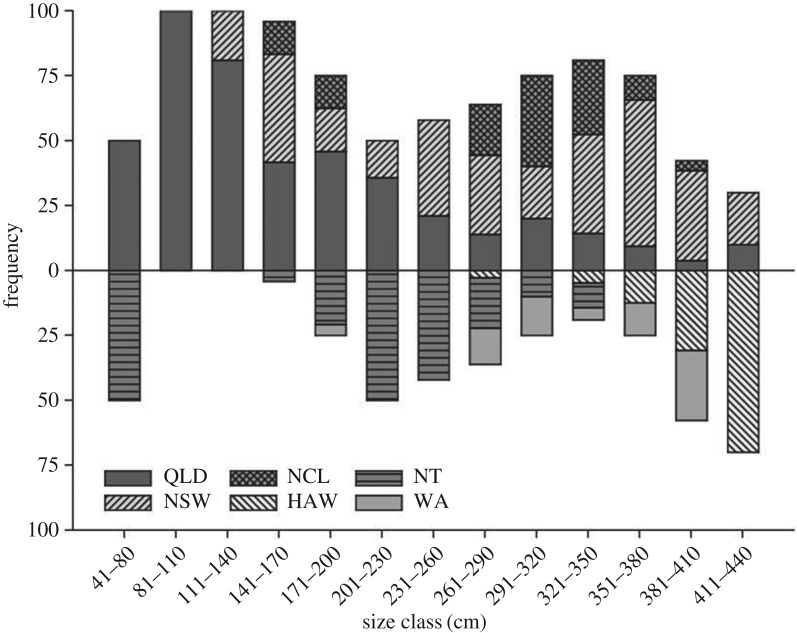
Length-frequency distribution of *G. cuvier* from which tissues were sampled in the Indo-Pacific region. No length measurements were recorded for Coral Sea samples.

Fin clips or muscle tissue were collected from *G. cuvier* caught in the western Pacific Ocean off the east coast of Australia (New South Wales, NSW; Queensland, QLD; the Coral Sea, COR; New Caledonia, NCL). Samples from the central Pacific Ocean were collected off Hawaii (HAW), while eastern Indian Ocean samples were obtained off Western Australia (WA), with additional northern Australian samples from the Northern Territory (NT). Out-group samples were obtained from Brazil (BRA) in the Atlantic Ocean (figure 1). All samples were collected between 2002 and 2015. As a guide to sample sizes required for the study, the theoretical statistical power required to detect genetic differentiation among populations was estimated using POWSIM [27]. Tissue was stored in 95% ethanol or 20% dimethyl sulfoxide (DMSO) solution. Genotypes were obtained using nine microsatellite loci previously developed for the species (see [26]). Sixty-nine juvenile (birth–200 cm TL), 69 sub-adult (200–300 cm TL), 96 adult (300 cm TL+), and 121 (size not recorded) *G. cuvier* (159 female, 107 male and 89 sex unknown) totalling 355 individuals were genotyped. Juvenile, sub-adult and adult sharks were sampled from QLD, NSW, NT and NCL, while predominantly adult sharks were sampled in WA and HAW (figure 2). Apart from during transit to Australia, all samples were stored at 4°C or below until laboratory processing.

DNA extraction was performed using either a QIAGEN DNeasy blood and tissue extraction kit following the manufacturer's protocols (QIAGEN Inc., Valencia, CA, USA), or a salting out method [28]. Loci were optimized into polymerase chain reaction (PCR) multiplexes using a fluorescently labelled M13 primer. For each locus, one primer stock consisting of forward and reverse primer pairs, and the corresponding M13 primer (Fam, Vic, Ned or Pet) was used. Primer stocks consisted of 3 µl of 100 µM forward primer, 30 µl of 100 µM reverse primer and 30 µl of 100 µM of M13-fluro labelled primer. To achieve optimal amplification across all loci, primer stock proportions were experimentally determined (see electronic supplementary material, table S1). Multiplex 1 consisted of loci tgr_1157, tgr_212, tgr_47, tgr_233 and tgr_348; and multiplex 2 consisted of tgr_1033, tgr_1185, tgr_891 and tgr_943. The samples were amplified using a 14 µl PCR mixture containing 10 ng of genomic DNA, 8 µl of master mix (1x Kapa Buffer A, 1.5% DMSO, 0.18 mM dNTP, 0.25 M Betaine, 0.8 units/reaction Taq), and 4 µl of primer mix (3 µl forward, 30 µl reverse, 30 µl M13-fluoro, 87 µl H_2_O). Loci were amplified in 2 multiplexes using the following protocols: Multiplex 1: 94°C for 2 min, followed by 12 cycles of 94°C for 15 s, 56°C for 30 s and 72°C for 45 s, with the annealing temperature reduced by 0.5°C each cycle (touchdown cycle). The reaction was then exposed to 23 cycles of 94°C for 15 s, 50°C for 30 s and 72°C for 45 s. Multiplex 2: 94°C for 2 min, followed by 10 cycles of 94°C for 15 s, 60°C for 30 s and 72°C for 45 s, reduced by 0.5°C each cycle. The reaction was then exposed to 23 cycles of 94°C for 15 s, 55°C for 30 s and 72°C for 45 s. The reactions for multiplex 1 and 2 completed at 72°C for 7 min before being held at 4°C until required for further analysis. Amplicons were diluted 60-fold, before being further diluted (1 in 5) in formamide containing LIZ-500 size standard (Applied Biosystems, Foster City, CA, USA) and then gel separated by capillary electrophoresis (Applied Biosystems 3130xl) following the manufacturer's recommendation.

### 2.2. Statistics

Alleles were sized against an internal size standard (GeneScan-500 LIZ) before being scored using GeneMapper v. 5. Genotypes were checked for scoring errors using Micro-Checker v. 2.2.3 [29]. We tested for Hardy–Weinberg equilibrium (HWE) using the Markov chain Monte Carlo (MCMC) method in GENEPOP v. 4.1.3 [30], with 100 000 steps, 100 batches and 10 000 subsequent iterations. We also tested for linkage disequilibrium (LD) among pairs of loci using an exact test based on a Markov chain method as implemented in GENEPOP, in both cases using Bonferroni to correct for multiple tests (*p* < 0.05) [31]. If significant departures from HWE or LD were found at one or more locus by population comparisons, then they were excluded from further analysis. To ensure that duplicate samples were not inadvertently included a Queller and Goodnight [32] relatedness test was performed in GenAlEx v. 6.5. Individuals that were identical across the nine microsatellite loci were treated as duplicates and excluded from further analysis.

Population pairwise standard diversity indices were estimated using GenAlEx v. 6.5, which included number of alleles (*N*_a_), number of effective alleles (*N*_e_), expected (*H*_e_) and observed heterozygosities (*H*_o_), and inbreeding coefficient (*F*_is_) [33]. To investigate the degree of subdivision between locations, pairwise *F*_ST_ values was estimated using Arlequin v. 3.11 [34] with the level of significance calculated by pseudo-replication (10,100) of individuals between locations; and Jost's *D*est (hereafter *D*est) was determined using GenAlEx v. 6.5 (999 iterations; [33,35]). Genetic populations were defined by locations (or groups of locations) that did not have significantly different *F*_ST_ values from one another, while showing significant variation to other sampled populations. An analysis of molecular variance (AMOVA) was also undertaken using a hierarchical approach in Arlequin v. 3.11, where population clusters were validated to ensure that the maximum amount of variance among groups of samples (regions) was maintained. Comparisons were made between the genotypes reported from Hawaii in Bernard *et al*. [9] and those herein to evaluate the presence of a separate Hawaiian population. To ensure the allele frequency bins were uniform between the studies at each locus, a comparison of dominant alleles was undertaken, and homology was determined for alleles having frequencies above 10% using allele sizes and per study frequencies. Two loci (tgr_348 and tgr_891) were unable to be reliably compared between the studies and were therefore excluded from the pairwise comparison analyses.

Bayesian cluster analysis was performed in STRUCTURE [36] on the microsatellite allele frequencies. STRUCTURE was run 10 independent times on the microsatellite dataset for the potential number of groups (*K*) ranging from *K* = 1 to 8. The run assumed an admixture ancestor model with a burn-in length of 100 000 MCMC steps, followed by simulation set at 1 000 000 repetitions. To estimate the number of groups (*K*) an *ad hoc* approach was taken by obtaining the mean posterior probability of the data Δ*K* using STRUCTURE Harvester [37]. To visualize clustering across runs of *K*, structure outputs were submitted to the CLUMPAK pipeline [38].

To identify the theoretical statistical power required to detect an *F*_ST_ differentiation among locations, pilot genotyping data from 96 randomly selected sharks was used in POWSIM [27]. Allele frequencies from the pilot data were used to simulate populations (differing by a range of user-defined overall *F*_ST_ values) to match populations sampled here from the three ocean basins (Central-West Pacific (CWP), Indo-East Indian (IEI), and the Atlantic). Three user-defined levels of divergence were simulated among populations; *F*_ST_ = 0.01, 0.005 and 0.0025. The following parameters were used to simulate the set of *F*_ST_ values: effective population sizes (*N*_e_) = 1000; number of simulations = 1000; and generations of drift (t) = 20 (*F*_ST_ = 0.01), 10 (*F*_ST_ = 0.005) and 5 (*F*_ST_ = 0.0025). To test for the effect of sample size on the ability to detect significant population differentiation (*α* = 0.05) at a given *F*_ST_ value, tests were undertaken with sample sizes of 25, 50 and 100 per population. The degree of significant differentiation between populations for each replicate run at a set *F*_ST_ value was tested using Chi-squared and Fisher's probabilities.

## 3. Results

### 3.1. Power simulation

When the power simulation was run with sample sizes of 100 across all nine microsatellite loci, there was sufficient power to delineate population subdivision in greater than 98% of simulations across the three levels of genetic differentiation tested (*F*_ST_ = 0.01, 0.005, 0.0025). When halving the sample size from 100 to 50, the ability to detect differences at the lowest *F*_ST_ (0.0025) decreased by 35%. Minor reductions in the ability to detect population structure (100% to more than 97%) were also found at *F*_ST_ = 0.005 and 0.01 when simulated sample sizes were reduced to 50. When sample sizes were further decreased to 25, an *F*_ST_ of 0.01 could be detected in over 95% of simulations; however, detections of smaller *F*_ST_ (from 0.005 and 0.0025; 58% and 22%, respectively) were considered unreliable. To summarize, the ability to detect differences among populations with low levels of genetic structure was found to decrease considerably with reductions in sample size.

### 3.2. Microsatellite diversity

Screening of the samples using a Queller and Goodnight [32] relatedness test detected 17 duplicates in the NCL samples, which were subsequently removed from further analysis. Across all samples, the nine loci had an average of 9.8 alleles (range 2–26) and unbiased heterozygosity of 0.70 (range 0.18–0.94). The most polymorphic locus was Tgr_891 (mean = 11.3 alleles), followed by Tgr_348 (9.7) and Tgr_943 (8.0), while Tgr_212 was the least polymorphic (1.4 alleles) (table 1). Screening of genotypes detected no linkage disequilibrium for any population, and genotype proportions at all loci did not deviate from Hardy–Weinberg expectations following Bonferroni correction. Pairwise *F*_ST_ and *D*est comparisons between locations indicated considerable genetic structuring between all Indo-Pacific locations and Brazil (*F*_ST_ values > 0.14, *p* < 0.001; *D*est values > 0.29, *p* < 0.001). In contrast, no significant genetic differences were observed between locations from within the Pacific or Indian Oceans (*F*_ST_ range 0.000–0.004; *D*est range 0.000–0.007) (table 2). As a result, collection locations within these regions were pooled for subsequent analysis by ocean basin. The ocean basin pools were the Central-West Pacific (CWP; consisting of NSW, QLD, COR, NCL, HAW), the Indo-East Indian (IEI; consisting of NT and WA) and the Atlantic Ocean (BRA). A comparison of *F*_ST_ and *D*est values among these three ocean basins was unable to identify any significant difference between CWP and IEI, which meant the null hypothesis of genetic homogeneity was unable to be rejected (table 3). Lack of genetic differentiation among samples from CWP and IEI was further supported by AMOVA analysis. Differences among groupings were maximized and variation within groupings was minimized when the Indo-Pacific samples (i.e. CWP and IEI) were considered as a single population (among groupings = 14.72%, within = 82.5%), compared to two separate populations (among groupings = 1.65%, within = 95.17%). Differentiation between the samples from Brazil (Atlantic) and all other locations were confirmed, with significant differences associated with grouped samples from the CWP (*F*_ST_ = 0.151, *p* < 0.001; *D*est = 0.321, *p* = 0.001) and IEI (*F*_ST_ = 0.145, *p* < 0.001; *D*est = 0.317, *p* = 0.001).

**Table 1.** Microsatellite marker diversity described by number of alleles (*N*_a_), number of effective alleles (*N*_e_), observed (*H*_o_) and expected heterozygosity (*H*_e_), and inbreeding coefficient (*F*_is_). *n* = number of individuals. Note that *n* in combined columns differs to total as not all loci could be genotyped per individual.

		Tgr_1033	Tgr_1157	Tgr_1185	Tgr_212	Tgr_233	Tgr_348	Tgr_47	Tgr_891	Tgr_943
QLD	*N*_a_	4	10	6	3	24	18	5	19	11
(*n*=69)	*N*_e_	1.964	6.283	3.582	1.646	7.081	9.338	1.808	12.253	8.056
	*H*_e_	0.441	0.750	0.706	0.348	0.912	0.924	0.485	0.939	0.853
	*H*_o_	0.491	0.841	0.721	0.392	0.859	0.893	0.447	0.918	0.876
	*F*_is_	0.101	0.108	0.021	0.114	−0.062	−0.035	−0.086	−0.023	0.026
NSW	*N*_a_	5	10	5	3	26	21	3	17	13
(*n*=81)	*N*_e_	2.275	5.717	3.125	1.391	7.772	12.189	1.547	11.685	8.258
	*H*_e_	0.560	0.825	0.680	0.281	0.871	0.918	0.354	0.914	0.879
	*H*_o_	0.605	0.838	0.738	0.309	0.888	0.911	0.300	0.901	0.877
	*F*_is_	−0.080	−0.015	−0.085	−0.098	−0.019	0.007	0.151	0.014	0.003
COR	*N*_a_	4	9	5	3	18	16	3	15	13
(*n*=36)	*N*_e_	2.185	6.234	3.871	1.536	6.863	9.138	1.885	11.187	9.554
	*H*_e_	0.556	0.800	0.781	0.361	0.886	0.912	0.417	0.943	0.853
	*H*_o_	0.542	0.840	0.742	0.349	0.854	0.891	0.470	0.911	0.895
	*F*_is_	−0.024	0.047	−0.053	−0.034	−0.037	−0.024	0.113	−0.035	0.047
NT	*N*_a_	4	10	6	3	24	21	4	17	12
(*n*=62)	*N*_e_	1.858	3.883	3.834	1.418	7.586	10.030	1.659	12.160	7.742
	*H*_e_	0.452	0.758	0.661	0.306	0.836	0.869	0.344	0.967	0.952
	*H*_o_	0.462	0.742	0.739	0.295	0.868	0.900	0.397	0.918	0.871
	*F*_is_	0.022	−0.021	0.105	−0.039	0.037	0.035	0.133	−0.054	−0.093
BRA	*N*_a_	3	5	4	2	9	9	2	12	8
(*n*=8)	*N*_e_	2.510	3.122	3.048	1.508	7.538	7.538	1.753	9.846	7.000
	*H*_e_	0.625	0.750	0.875	0.429	0.857	1.000	0.625	0.875	1.000
	*H*_o_	0.602	0.680	0.672	0.337	0.867	0.867	0.430	0.898	0.857
	*F*_is_	−0.039	−0.103	−0.302	−0.273	0.012	−0.153	−0.455	0.026	−0.167
NCL	*N*_a_	4	8	6	3	15	15	3	13	10
(*n*=22)	*N*_e_	1.699	4.500	3.868	1.340	7.056	8.647	2.066	10.127	7.670
	*H*_e_	0.400	0.810	0.810	0.286	0.952	0.857	0.476	0.850	0.952
	*H*_o_	0.411	0.778	0.741	0.254	0.858	0.884	0.516	0.901	0.870
	*F*_is_	0.027	−0.041	−0.092	−0.125	−0.110	0.031	0.077	0.057	−0.095
WA	*N*_a_	5	9	6	4	22	19	3	17	12
(*n*=56)	*N*_e_	2.047	4.938	3.269	1.524	8.267	11.521	1.863	12.398	6.938
	*H*_e_	0.526	0.807	0.649	0.298	0.930	0.895	0.439	0.982	0.839
	*H*_o_	0.512	0.797	0.694	0.344	0.879	0.913	0.463	0.919	0.856
	*F*_is_	−0.029	−0.012	0.065	0.133	−0.058	0.020	0.053	−0.068	0.019
HAW	*N*_a_	7	10	4	3	14	13	4	15	12
(*n*=21)	*N*_e_	1.869	5.765	3.139	1.156	5.345	9.660	2.353	11.025	8.791
	*H*_e_	0.571	0.667	0.579	0.095	0.619	0.938	0.550	0.905	0.950
	*H*_o_	0.465	0.827	0.681	0.135	0.813	0.896	0.575	0.909	0.886
	*F*_is_	−0.229	0.193	0.150	0.294	0.238	−0.046	0.043	0.005	−0.072
Combined	*n*	353	352	347	354	350	341	351	347	349
(*n*=355)	*Mean N*_a_	4.500	8.875	5.250	3.000	19.000	16.500	3.375	15.625	11.375
	*Mean F*_is_	−0.032	0.024	−0.023	−0.019	−0.001	−0.020	0.004	−0.010	−0.041

**Table 2.** Pairwise *F*_ST_ values (above diagonal) and *D*est values (below diagonal) for *G. cuvier* across eight sampling locations. Significant results were accepted at *p* < 0.01 and denoted by *, with *p*-value listed in parentheses.

	QLD	NSW	COR	NT	NCL	WA	HAW	BRA
QLD	—	−0.001	−0.008	−0.002	0.003	0.003	0.001	0.146*
		(0.820)	(0.766)	(0.891)	(0.216)	(0.054)	(0.405)	(0.000)
NSW	−0.003	—	−0.003	0.000	0.003	0.003	−0.004	0.152*
	(0.808)		(0.891)	(0.487)	(0.261)	(0.036)	(0.847)	(0.000)
COR	−0.003	−0.007	—	0.002	0.002	0.000	0.001	0.167*
	(0.631)	(0.926)		(0.225)	(0.252)	(0.451)	(0.559)	(0.000)
NT	−0.006	−0.001	0.003	—	0.004	0.002	0.000	0.143*
	(0.944)	(0.644)	(0.306)		(0.162)	(0.190)	(0.541)	(0.000)
NCL	0.005	0.005	0.001	0.006	—	0.000	0.001	0.167*
	(0.276)	(0.237)	(0.457)	(0.218)		(0.423)	(0.441)	(0.000)
WA	0.007	0.007	−0.001	0.003	0.001	—	−0.001	0.151*
	(0.076)	(0.053)	(0.529)	(0.243)	(0.437)		(0.514)	(0.000)
HAW	0.002	−0.008	0.000	−0.002	0.000	−0.001	—	0.157*
	(0.396)	(0.873)	(0.436)	(0.561)	(0.434)	(0.507)		(0.000)
BRA	0.321*	0.32*	0.336*	0.319*	0.331*	0.315*	0.298*	—
	(0.001)	(0.001)	(0.001)	(0.001)	(0.001)	(0.001)	(0.001)

**Table 3.** Pairwise *F*_ST_ values (above diagonal) and *D*est values (below diagonal) for *G. cuvier* across three grouped sampling locations. Significant results were accepted at *p* < 0.01 and denoted by *, with *p*-value listed in parentheses.

	Central-West Pacific	Indo-East Indian	Atlantic
Central-West Pacific	—	0.000 (0.135)	0.151* (0.000)
Indo-East Indian	0.002 (0.192)	—	0.145* (0.000)
Atlantic	0.321* (0.001)	0.317* (0.001)	—

Pairwise comparisons were also made between the Hawaiian genotypes from this study, and the Central Pacific (CP) genotypes reported in Bernard *et al*. [9]. No significant differences were observed between Hawaiian samples from both datasets. The similarity between datasets was reflected in the allele frequency distributions between datasets (see electronic supplementary material).

Bayesian STRUCTURE analysis of microsatellite genotypes supported the groupings identified by *F*_ST_. The Δ*K* method in STRUCTURE Harvester identified the *K* = 2 as the most likely number of populations. Visual clustering of individuals using CLUMPAK revealed that all samples from the Indian and Pacific Ocean were assigned to the same group, while the second group was made up of samples from Brazil. To investigate the Bayesian structuring of individuals within the Indo-Pacific, samples from Brazil were removed before the remaining samples were re-analysed. When subsequent STRUCTURE analysis including only the Indo-Pacific samples was run, the Δ*K* suggested only one discernible grouping was present within the data.

## 4. Discussion

Analysis of genetic homogeneity with microsatellites across all locations in this study revealed two genetically independent populations; an Indo-Pacific group composed of all samples collected in the IEI and the CWP, and the Brazilian sample from the western Atlantic. The absence of genetic differentiation between the east and west Australian coasts is consistent with previously reported movement data, with evidence of migrating sharks from both coasts moving into adjoining Northern Territorial waters (see [18,19]). Evidence of tiger shark movement through the Torres Strait land bridge [18] suggests that the relatively shallow waters (less than 15 m deep between Cape York, QLD and Papua New Guinea) do not act as a barrier to movements, and possibly gene flow, as it does for other fish species [39]. A high level of genetic connectivity throughout the Indo-Pacific region was also reported by Bernard *et al*. [9] in their global study, with little differentiation observed between sharks sampled from the east and west Australian coasts, the Andaman Sea (southeast Asia) and as far west as the eastern seaboard of South Africa.

Our investigation suggests genetic connectivity among tiger sharks from the Indo-Pacific extends into the Pacific Ocean as far as Hawaii (central Pacific). This finding was in contrast to that identified in the global study by Bernard *et al*. [9], which reported significant differences between Hawaii and all other global sites surveyed, including those from the Indo-Pacific. The STRUCTURE analysis by Bernard *et al*. [9] may explain the differences observed between the two studies. Despite Δ*K* suggesting *K* = 2 throughout all global samples, when *K* = 3 was assumed by Bernard *et al*. [9], a few individuals from the southwest Pacific (eastern Australia) assigned to a third cluster, which was subsequently reported as evidence of a genetically distinct Hawaiian population. Although Bernard *et al*. [9] demonstrated a weak signal for population subdivision comparing samples from Eastern Australia (*n* = 21) and Hawaii (*n* = 65) (*F*_ST_ = 0.01, *D*est = 0.02), the low statistical power due to the small sample size from Eastern Australia reduces the likelihood that the statistically significant finding actually reflects a true effect [40]. Moreover, the power analysis completed herein demonstrates that the ability to detect differences among tiger shark populations with low levels of genetic structure was found to decrease considerably with reductions in sample size less than 25. Ecological studies undertaking satellite tracking across the Hawaiian archipelago have shown that tiger sharks in this region do exhibit regionally localized movements (e.g. [21,23,41]). Notwithstanding, there is a confirmed record of a conventionally tagged tiger shark from Hawaii being captured in Mexico, approximately 5000 km east of its tagging location (C. Meyer 2016, personal communication). This recapture indicates that tiger sharks have the ability to cross the large oceanic expanses, with the possibility of promoting gene flow across the broader Pacific Ocean. Acquiring and analysing additional tissue samples from the eastern Pacific region, along with further research using alternative genetic markers such as mtDNA and single nucleotide polymorphisms (SNP) in nuclear DNA is needed to confirm the single population. The level of heterozygosity of microsatellite loci reported herein suggests that variation in SNP loci should also be high. The development of SNPs for *G. cuvier* is likely to increase the power to detect genetic variation, even if sample sizes are small [42].

Continued advances in tagging technology that allow for multi-year data collection from mature sharks, and further developments in molecular analyses, is likely to improve our understanding of how partial migration, sex-biased dispersal and reproductive mixing influences population structure and connectivity of tiger sharks within and among global ocean basins. Given the evidence of exploitation-driven declines in *G. cuvier* across the world [14,15,43,44], continued assessments are vital to inform population-level management and conservation efforts across the entire distribution of this oceanodromous species.

## Supplementary Material

Figure S1

## Supplementary Material

Figure S2

## Supplementary Material

Table S1

## Data accessibility

Tiger shark genotypes are available through the UQ eSpace (http://espace.library.uq.edu.au/view/UQ:613874).

## References

[1] Fowler S 2014 The conservation status of migratory sharks, 30pp Bonn, Germany: UNEP/CMS Secretariat.

[2] Geraghty PT, Williamson JE, Macbeth WG, Wintner SP, Harry AV, Ovenden JR, Gillings MR 2013 Population expansion and genetic structure in *Carcharhinus brevipinna* in the Southern Indo-Pacific. PLoS ONE 9, e94738 (doi:10.1371/journal.pone.0075169) 10.1371/journal.pone.0075169 PMC3783459 24086462

[3] Ashe JL, Feldheim KA, Fields AT, Reyier EA, Brooks EJ, O'Connell MT, Skomal G, Gruber SH, Chapman DD 2015 Local population structure and context-dependent isolation by distance in a large coastal shark. Mar. Ecol. Prog. Ser. 520, 203–216. (doi:10.3357/meps11069)

[4] Palumbi SR 1994 Genetic divergence, reproductive isolation and marine speciation. Annu. Rev. Ecol. Syst. 25, 547–572. (doi:10.1146/annurev.es.25.110194.002555)

[5] DeWoody JA, Avise JC 2000 Microsatellite variation in marine, freshwater and anadromous fishes compared with other animals. J. Fish Biol. 56, 461–473. (doi:10.1111/j.1095-8649.2000.tb00748.x)

[6] Benavides MT et al. 2011 Global phylogeography of the dusky shark *Carcharhinus obscurus*: implications for fisheries management and monitoring the shark fin trade. Endangered Species Res. 14, 13–22. (doi:10.3354/esr00337)

[7] Blower DC, Pandolfi JM, Bruce BD, Gomez-Cabrera MdC, Ovenden JR 2012 Population genetics of Australian white sharks reveals fine-scale spatial structure, transoceanic dispersal events and low effective population sizes. Mar. Ecol. Prog. Ser. 455, 229–244. (doi:10.3354/meps09659)

[8] Dudgeon CL et al. 2012 A review of the application of molecular genetics for fisheries management and conservation of sharks and rays. J. Fish Biol. 80, 1789–1843. (doi:10.1111/j.1095-8649.2012.03265.x) 22497408 10.1111/j.1095-8649.2012.03265.x

[9] Bernard AM, Feldheim KA, Heithaus MR, Wintner SP, Wetherbee BM, Shivji MS 2016 Global population genetic dynamics of a highly migratory, apex predator shark. Mol. Ecol. 25, 5312–5329. (doi:10.1111/mec.13845) 27662523 10.1111/mec.13845

[10] Pepperell JG 1992 Trends in the distribution, species composition and size of sharks caught by gamefish anglers off south-eastern Australia, 1961–90. Aust. J. Mar. Freshw. Res. 43, 213–225. (doi:10.1071/MF9920213)

[11] Wetherbee BM, Lowe CG, Crow GL 1994 A review of shark control in Hawaii with recommendations for future research. Pac. Sci. 48, 95–115. (doi:hdl.handle.net/10125/2202)

[12] Macbeth WG, Geraghty PT, Peddemors VM, Gray CA 2009 *Observer-based study of targeted commercial fishing for large shark species in waters off northern New South Wales: final report to the Northern Rivers Catchment Management Authority, Project No. IS8-9-M-2*, Fisheries Final Report Series 114. Cronulla, NSW, Australia: Industry and Investment NSW.

[13] Liston J, Clark G, Alexander D 2011 Pacific island heritage: archaeology, identity and community. Canberra, ACT, Australia: ANU E Press, The Australian National University.

[14] Reid DD, Robbins WD, Peddemors VM 2011 Decadal trends in shark catches and effort from the New South Wales Shark Meshing Program 1950 to 2010. Mar. Freshw. Res. 62, 676–693. (doi:10.1071/MF10162)

[15] Holmes BJ, Sumpton WD, Mayer DG, Tibbetts IR, Neil DT, Bennett MB 2012 Declining trends in annual catch rates of the tiger shark (*Galeocerdo cuvier*) in Queensland, Australia. Fish. Res. 129–130, 38–45. (doi:dx.doi.org/10.1016/j.fishres.2012.06.005)

[16] Holmes BJ, Pepperell JP, Griffiths SP, Jaine FRA, Tibbetts IR, Bennett MB 2014 Tiger shark (*Galeocerdo cuvier*) movement patterns and habitat use determined by satellite tagging in eastern Australian waters. Mar. Biol. 161, 2645–2658.

[17] Werry JM, Planes S, Berumen ML, Lee KA, Braun CD, Clua E 2014 Reef-fidelity and migration of tiger sharks, *Galeocerdo cuvier*, across the Coral Sea. PLoS ONE 9, e83249 (doi:10.1371/journal.pone.0083249) 24421879 10.1371/journal.pone.0083249 PMC3885424

[18] Fitzpatrick R, Thums M, Bell I, Meekan M, Stevens JD, Barnett A 2012 A comparison of the seasonal movements of tiger sharks and green turtles provides insights into their predator-prey relationship. PLoS ONE 7, e51927 (doi:10.1371/journal.pone.0051927) 23284819 10.1371/journal.pone.0051927 PMC3526478

[19] Ferreira LC, Thums M, Meeuwig JJ, Vianna GMS, Stevens J, McAuley R, Meekan MG 2015 Crossing latitudes—long-distance tracking of an apex predator. PLoS ONE 10, e0116916 (doi:10.1371/journal.pone.0116916) 25671609 10.1371/journal.pone.0116916 PMC4324986

[20] Heithaus MR, Wirsing AJ, Dill LM, Heithaus LI 2007 Long-term movements of tiger sharks satellite-tagged in Shark Bay, Western Australia. Mar. Biol. 151, 1455–1461. (doi:10.1007/s00227-006-0583-y)

[21] Holland KN, Wetherbee BM, Lowe CG, Meyer CG 1999 Movements of tiger sharks (*Galeocerdo cuvier*) in coastal Hawaiian waters. Mar. Biol. 134, 665–673. (doi:10.1007/s002270050582)

[22] Meyer CG, Clark TB, Papastamatiou YP, Whitney NM, Holland KN 2009 Long-term movement patterns of tiger sharks *Galeocerdo cuvier* in Hawaii. Mar. Ecol. Prog. Ser. 381, 223–235. (doi:10.3354/meps07951)

[23] Papastamatiou YP, Meyer CG, Carvalho F, Dale JJ, Hutchinson MR, Holland KN 2013 Telemetry and random walk models reveal complex patterns of partial migration in a large marine predator. Ecol. Soc. Am. 94, 2595–2606. (doi:10.1890/12-2014.1) 10.1890/12-2014.1 24400511

[24] Whitney NM, Crow GL 2007 Reproductive biology of the tiger shark (*Galeocerdo cuvier*) in Hawaii. Mar. Biol. 151, 63–70. (doi:10.1007/s00227-006-0476-0)

[25] Dulvy NK et al. 2008 You can swim but you can't hide: the global status and conservation of oceanic pelagic sharks and rays. Aquat. Conserv. 18, 459–482. (doi:10.1002/aqc.975)

[26] Bernard AM, Feldheim KA, Shivji MS 2015 Isolation and characterization of polymorphic microsatellite markers from a globally distributed marine apex predator, the tiger shark (*Galeocerdo cuvier*). Conserv. Genet. Resour. 7, 509–511. (doi:10.1007/s12686-014-0408-0)

[27] Ryman N, Palm S 2006 POWSIM: a computer program for assessing statistical power when testing for genetic differentiation. Mol. Ecol. Notes 6, 600–602. (doi:10.1111/j.1471-8286.2006.01378.x) 10.1046/j.0962-1083.2001.01345.x 11703649

[28] Sunnucks P, Hales DF 1996 Numerous transposed sequences of mitochondrial cytochrome oxidase I-II in aphids of the genus *Sitobion* (Hemiptera: Aphididae). Mol. Biol. Evol. 13, 510–524. (doi:10.1093/oxfordjournals.molbev.a025612) 8742640 10.1093/oxfordjournals.molbev.a025612

[29] Van Oosterhout C, Hutchinson W, Wills D, Shipley P 2004 Microchecker: software for identifying and correcting genotyping errors in microsatellite data. Mol. Ecol. Notes 4, 535–538. (doi:10.1111/j.1471-8286.2004.00684.x)

[30] Raymond M, Rousset F 1995 An exact test for population differentiation. Evolution 49, 1280–1283. (doi:10.2307/2410454) 28568523 10.1111/j.1558-5646.1995.tb04456.x

[31] Rice WR 1989 Analysing tables of statistical tests. Evolution 43, 223–225. (doi:10.2307/2409177) 28568501 10.1111/j.1558-5646.1989.tb04220.x

[32] Queller DC, Goodnight KF 1989 Estimating relatedness using genetic markers. Evolution 43, 258–275. (doi:10.2307/2409206) 28568555 10.1111/j.1558-5646.1989.tb04226.x

[33] Peakall R, Smouse PE 2012 GenAlEx 6.5: genetic analysis in Excel. Population genetic software for teaching and research—an update. Bioinformatics 28, 2537–2539. (doi:10.1093/bioinformatics/bts460) 22820204 10.1093/bioinformatics/bts460 PMC3463245

[34] Excoffier L, Laval G, Schneider S 2005 Arlequin (version 3.0): an integrated software package for population genetics data analysis. Evol. Bioinformatics Online 1, 47–50. PMC2658868 19325852

[35] Jost L 2008 G_ST_ and its relatives do not measure differentiation. Mol. Ecol. 17, 4015–4026. (doi:10.1111/j.1365-294X.2008.03887.x) 19238703 10.1111/j.1365-294x.2008.03887.x

[36] Pritchard JK, Stephens M, Donnelly P 2000 Inference of population structure using multilocus genotype data. Genetics 155, 945–959. 10835412 10.1093/genetics/155.2.945 PMC1461096

[37] Earl DA, Vonholdt BM 2012 STRUCTURE HARVESTER: a website and program for visualizing STRUCTURE output and implementing the Evanno method. Conserv. Genet. Resour. 4, 359–361. (doi:10.1007/s12686-011-9548-7)

[38] Kopelman NM, Mayzel J, Jakobsson M, Rosenberg NA, Mayrose I 2015 Clumpak: a program for identifying clustering modes and packaging population structure inferences across K. Mol. Ecol. Resour. 15, 1179–1191. (doi:10.1111/1755-0998.12387) 25684545 10.1111/1755-0998.12387 PMC4534335

[39] Mirams AGK, Tremi EA, Shields JL, Liggins L, Riginos C 2011 Vicariance and dispersal across an intermittent barrier: population genetic structure of marine animals across the Torres Strait land bridge. Coral Reefs 30, 937–949. (doi:10.1007/s00338-011-0767-x)

[40] Button KS, Loannidis JPA, Mokrysz C, Nosek BA, Flint J, Robinson ESJ, Munafó MR 2013 Power failure: why small sample size undermines the reliability of neuroscience. Nat. Rev. Neurosci. 14, 365–376. (doi:10.1038/nrn3475) 23571845 10.1038/nrn3475

[41] Meyer CG, Papastamatiou YP, Holland KN 2010 A multiple instrument approach to quantifying the movement patterns and habitat use of tiger (*Galeocerdo cuvier*) and Galapagos sharks (*Carcharhinus galapagensis*) at French Frigate Shoals, Hawaii. Mar. Biol. 157, 1857–1868. (doi:10.1007/s00227-010-1457-x)

[42] Willing E, Dreyer C, van Oosterhout C 2012 Estimates of genetic differentiation measured by F_ST_ do not necessarily require large sample sizes when using many SNP markers. PLoS ONE 7, e42649 (doi:10.1371/journal.pone.0042649) 22905157 10.1371/journal.pone.0042649 PMC3419229

[43] Baum JK, Myers RA, Kehler DG, Worm B, Harley SJ, Doherty PA 2003 Collapse and conservation of shark populations in the northwest Atlantic. Science 299, 389–392. (doi:10.1126/science.1079777) 12532016 10.1126/science.1079777

[44] Myers RA, Baum JK, Shepherd TD, Powers SP, Peterson CH 2007 Cascading effects of the loss of apex predatory sharks from a coastal ocean. Science 315, 1846–1850. (doi:10.1126/science.1138657) 17395829 10.1126/science.1138657

