# Supplementary figures and images for: Population structure and connectivity of tiger sharks (Galeocerdo cuvier) across the Indo-Pacific Ocean basin
Source: R Soc Open Sci. 2017 Jul 5;4(7):170309. doi: 10.1098/rsos.170309 (PMC5541554; doi:10.1098/rsos.170309)

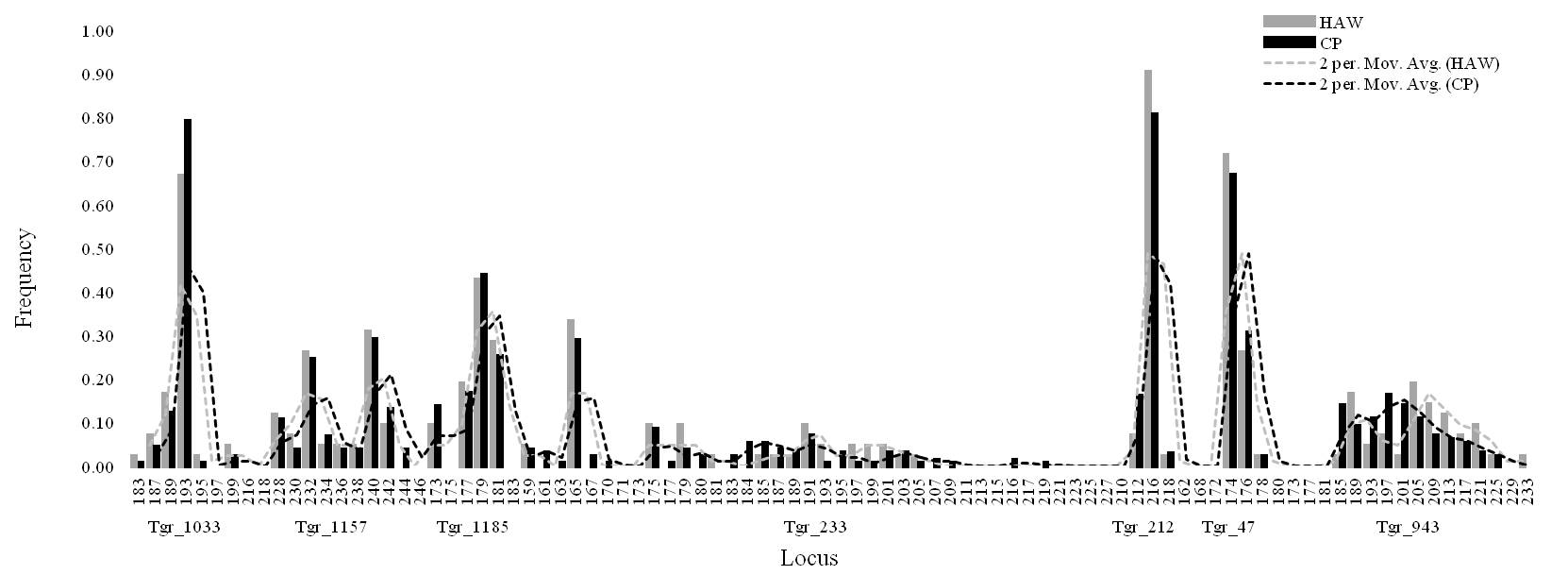

Supplement: Figure S1 [file rsos170309supp1.jpg]

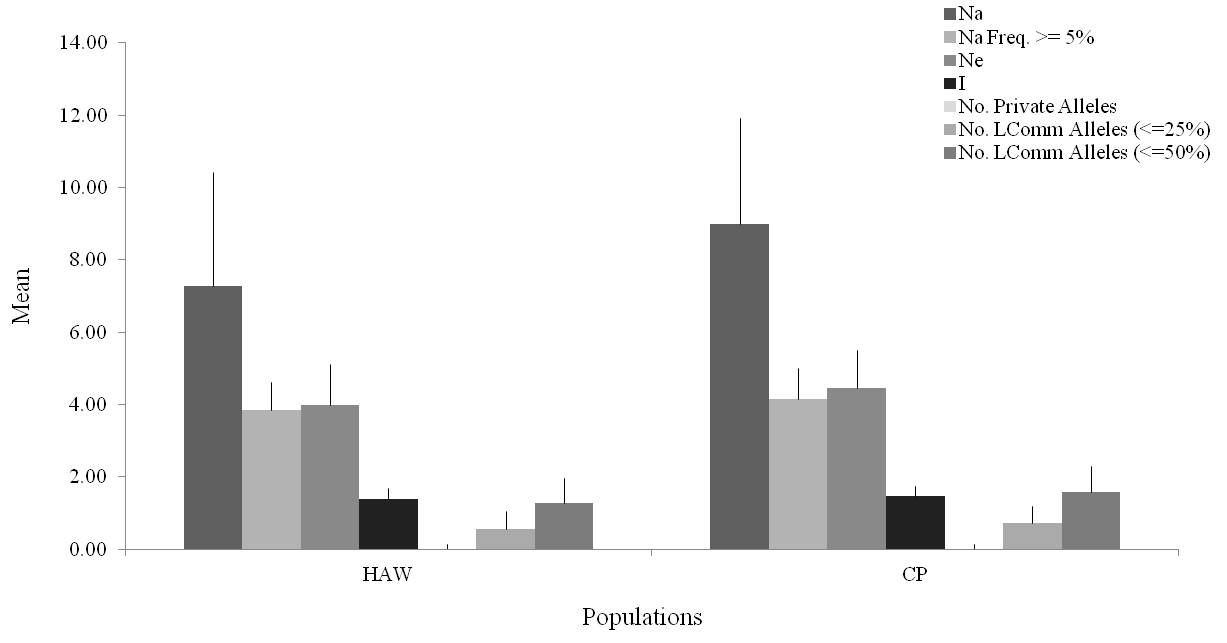

Supplement: Figure S2 [file rsos170309supp2.jpg]

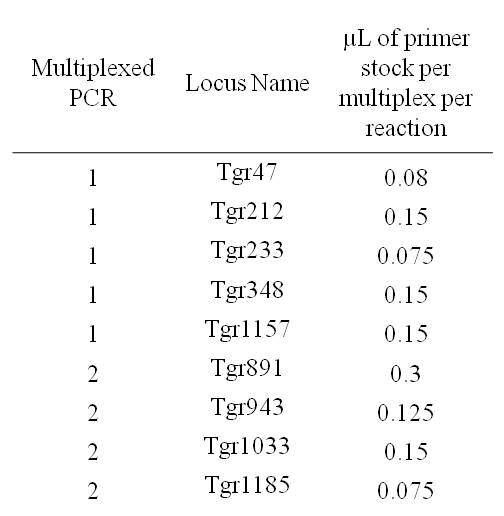

Supplement: Table S1 [file rsos170309supp3.jpg]
